# K-paracelsian (KAlSi_3_O_8_·H_2_O) and identification of a simple building scheme of dense double-crankshaft zeolite topologies

**DOI:** 10.1107/S2052252518016111

**Published:** 2019-01-01

**Authors:** Cristian-R. Boruntea, Peter N. R. Vennestrøm, Lars F. Lundegaard

**Affiliations:** a Haldor Topsøe A/S, Haldor Topsøes Alle 1, Kgs. Lyngby 2800, Denmark; b Instituto de Tecnología Química, Universitat Politècnica de València-Consejo Superior de Investigaciones Científicas, Avenida de los Naranjos s/n, Valencia 46022, Spain; c Umicore Denmark ApS, Nøjsomhedsvej 20, Kgs. Lyngby 2800, Denmark

**Keywords:** K-paracelsian, dense double-crankshaft zeolite topologies, structure refinement, crystal engineering, inorganic porous solids, microporous materials

## Abstract

During screening of the phase space using KOH and 1-methyl-4-aza-1-azoniabicyclo­[2.2.2]octane hydroxide (1-methyl-DABCO) under hydrothermal zeolite synthesis conditions, K-paracelsian was synthesized. Scanning electron microscopy, energy dispersive X-ray spectroscopy and *ex situ* powder X-ray diffraction analysis revealed a material that is compositionally closely related to the mineral microcline and structurally closely related to the mineral paracelsian, both of which are feldspars. In contrast to the feldspars, K-paracelsian contains intrazeolitic water corresponding to one molecule per cage. In the case of K-paracelsian it might be useful to consider it a link between feldspars and zeolites. It was also shown that K-paracelsian can be described as the simplest endmember of a family of dense double-crankshaft zeolite topologies. By applying the identified building principle, a number of known zeolite topologies can be constructed. Furthermore, it facilitates the construction of a range of hypothetical small-pore structures that are crystallo-chemically healthy, but which have not yet been realized experimentally.

## Abstract 2

The synthesis and structure refinement of a dense zeolite with a one-dimensional channel system is reported.

## 1. Introduction   

Zeolites are microporous aluminosilicates belonging to the tectosilicates group, characterized by a well defined and uniform pore system, free channels and internal voids where water can be located. Their crystalline structure contains tetrahedrally coordinated Si and Al that are connected through oxygen atoms, forming a three-dimensional network with cavities of different shapes and sizes that can host various cations. These structural features, in combination with the hosted cation, are in direct correlation with their properties that are highly exploited in catalysis, ion exchange and separation applications (Corma, 1995 ▸; Davis, 2002 ▸).

Synthetic zeolites were initially prepared by mimicking the conditions under which zeolites are formed in nature: aqueous inorganic alkaline solutions were combined with sources of silica and alumina and treated under high pressure (Barrer, 1982 ▸). Due to the need for more siliceous materials, most novel zeolites are synthesized today by using organic cations that are soluble in aqueous media, acting as organic structure-directing agents (OSDAs), combined with the necessary reaction conditions and a mineralizing agent that allows the nucleation and crystal growth of the desired structure. It is generally accepted that the organic molecules used during the synthesis are incorporated into the zeolite cavities, stabilizing the structure through van der Waals interactions, among others. However, there are examples where the organic molecule is not occluded, but either participates in or modifies the zeolite crystallization and/or growth mechanism. For example, the synthesis of Losod (LOS), a new framework structure at the time (Sieber & Meier, 1974 ▸), could be prepared without an OSDA being incorporated into the zeolite cavities. In this case, it was speculated that the organic cations might be responsible for keeping the aluminate and silicate species in a reactive state, suggesting that the OSDA is playing a catalytic role rather than having a classic structure-directing effect. Furthermore, it was shown that the synthesis of Losod was highly dependent on the use of sodium cations, which were found to be part of the zeolite product (Sieber & Meier, 1974 ▸). This is a good example of how an organic molecule can influence product selectivity, while at the same time the inorganic cation becomes incorporated in the final material. Both cations seem to play a concerted role in this type of crystallization.

In this contribution we report on the synthesis and structure refinement of the novel dense zeolite material K-paracelsian with a one-dimensional channel system. The zeolite was discovered while screening the hydro­thermal phase space using potassium hydroxide in combination with 1-methyl-4-aza-1-azoniabi­cyclo­[2.2.2]octane hydroxide (1-methyl-DABCO) and the use of a FAU zeolite as the source of Si and Al. We found that the novel synthesized aluminosilicate is compositionally closely related to the mineral microcline and structurally closely related to the mineral paracelsian. Both are part of the feldspar group. In contrast to feldspars, this new material shows intrazeolitic molecular water, which can be extracted upon heating. We also show that K-paracelsian is the simplest endmember of a family of dense double-crankshaft zeolite topologies.

## 2. Experimental   

### 2.1. Synthesis   

For a typical synthesis, 2.32 g 1-methyl-DABCO (12.5 wt%) was mixed with 1.97 g KOH (20 wt%) and 0.743 g H-FAU (Si/Al = 6) to give a molar composition of 1(Si): 0.167(Al): 0.2(OSDA): 0.7(KOH): 20(H_2_O). The resulting gel was mixed for 1 h at room temperature and then transferred into a Teflon lined autoclave. The crystallization was carried out at 150°C for 7 days unless otherwise stated. Solid yields are calculated based on Si/Al ratio in the reactant compared with the product. The raw materials used included: 1-methyl-DABCO prepared in-house (see supporting information for the synthesis procedure), KOH (reagent grade ≥98%, Sigma–Aldrich), in-house prepared co-precipitated amorphous silica–alumina (ASA) and Y-zeolite (CBV-712, from Zeolyst International).

### 2.2. Characterization   

The synthesized materials were analyzed by X-ray diffraction using a Phillips PW1800 instrument system in θ–2θ geometry working in Bragg–Brentano mode using Cu *K*α radiation (λ = 1.541 Å). An AntonPaar1200 cell equipped with a capillary stage was used for the *in situ* X-ray diffraction (XRD) heating experiments. Scanning electron microscopy (SEM) images were recorded using an XL30-SEM instrument operated at 10 kV. The samples were sprinkled over carbon tape, attached to Al stubs and then coated with a 30 nm layer of carbon to prevent charging. Thermogravimetric analysis (TGA) and differential scanning calorimetry (DSC) were performed using a Mettler TGA–DSC instrument with a heating rate of 5°C min^−1^ and a temperature limit of 550°C. One-dimensional ^13^C-NMR was measured using a Brukner 400 MHz instrument in D_2_O, and for the solid-state ^13^C CP-MAS NMR, the spectra were acquired at 9.4 T using a spinning speed of *nR* = 10.0 kHz and a CP contact time of 5.0 ms. For the ^27^Al and ^29^Si MAS NMR spectra, a Bruker Avance II HD 400 MHz spectrometer was used. ^27^Al MAS NMR spectra were recorded at spinning rate of 20 kHz on a Bruker 3.2 mm probe, using a flip angle of π/12 and a recycle delay of 1 s. ^29^Si MAS NMR spectra were recorded at a spinning rate of 5 kHz on a Bruker 4 mm probe using a flip angle of 60° and a recycle delay of 60 s.

## 3. Results and discussion   

### 3.1. Synthesis   

The phase space using 1-methyl-DABCO was explored in alkaline media using NaOH or KOH. Two sources of *T* atoms (Si and Al) were selected for this screening study, namely the use of a co-precipitated silica–alumina as well as FAU. When 1-methyl-DABCO is used in combination with NaOH and FAU no crystalline products are observed at low OH^−^ concentrations. Increasing the OH^−^ concentration leads to a narrow region with LEV in line with previous studies (Xie *et al.*, 2013 ▸). Further increase of the basicity yields ANA as the thermodynamic end-point in Na-rich systems (see Table S1).

In agreement with the example of LOS mentioned in the introduction and many other studies, exchanging the inorganic cation leads to a new set of obtainable phases. At low OH^−^ concentrations and high silica compositions (*e.g.* Si/Al gel ratios ≥ 15), when potassium hydroxide is applied together with the 1-methyl-DABCO organic molecule, a phase that we have not yet identified appears together with ERI, irrespective of the *T* atom source (see Table S2). At higher OH^−^ concentrations and in more Al-rich synthesis gels (Si/Al gel ratio = 6), phase selectivity becomes dependent on the *T* atom source (see Table 1 ▸). Using an amorphous co-precipitated silica–alumina leads to the well known LTL phase. However, exchanging the *T* atom source to a FAU zeolite leads to another zeolite product (named K-paracelsian herein, see below). This zeolite is furthermore dependent on the concerted effect of both 1-methyl-DABCO and potassium hydroxide. This is evident because the phase is only present as a small impurity when 1-methyl-DABCO is omitted in the gel (see sample 5 in Table 1 ▸). We also note that ERI is seen at lower hydroxide concentrations together with an unknown phase.

**Figure 1:**
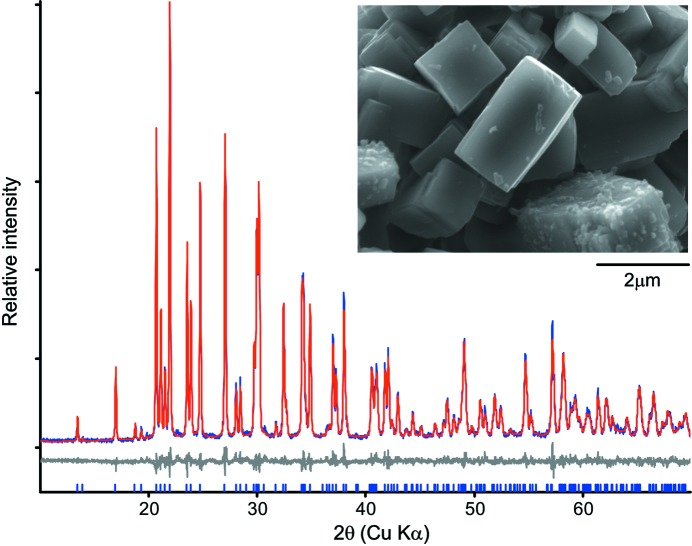
Powder X-ray diffraction pattern and SEM image (insert) of K-paracelsian. The blue curve is the observed data, the red curve is the Rietveld fit of the final structure model and the grey curve is the difference curve.

A phase-pure sample of the new zeolite was achieved (sample 4 in Table 1 ▸ and Fig. 1 ▸) by combination of high OH^−^ concentrations, an FAU as a *T* atom source and the combination of potassium hydroxide and 1-methyl-DABCO. Occlusion of the OSDA in the zeolite could not easily be measured by ^13^C CP-MAS NMR. Only when the number of scans was increased threefold could a small amount of C be measured in the sample (Fig. S1). This is consistent with the interpretation that 1-methyl-DABCO is not occluded in the pore system, which would have been expected from the classical role of an OSDA, but a small amount can be found on the surface of the crystallites. In the remaining part of the manuscript, we will focus on this material specifically.

### 3.2. Basic characterization and structure refinement   

The initial characterization used to reveal the structure of the product (sample 4, Table 1 ▸) consisted of SEM imaging and *ex situ* XRD analysis of the as-synthesized products. SEM images revealed a homogeneous morphology consisting of large (∼2 µm) well faceted crystallites. Point EDS analysis on five different crystallites revealed an average composition of 3.3 (3) Si/Al and 0.29 (3) K/Si. Within the uncertainty of the EDS analysis, this is consistent with a stoichiometric compound of the composition KAlSi_3_O_8_, which is also in agreement with the expected Si/Al ratio from the yield reported in Table 1 ▸ and the Si/Al ratio of 2.92 calculated from ^27^Si MAS NMR (Fig. S2).

Several attempts using the search–match software *HighScorePlus* combined with the PDF4+ database (Kabekkodu, 2018 ▸) did not result in identification of the phase. Because the search–match routine failed, an attempt to index the observed powder pattern was performed. Indexing and subsequent LeBail fitting (Coelho, 2018 ▸) revealed an orthorhombic unit cell, with the dimensions *a* = 9.198 (2) Å, *b* = 9.476 (2) Å and *c* = 8.593 (2) Å. The orthorhombic symmetry is also consistent with the morphology observed in the SEM images (see Fig. 1 ▸). A search of the ICSD (Hellenbrandt, 2004 ▸) using the observed lattice parameters with a 2% tolerance resulted in a match with four different published structures of paracelsian. This is a feldspar mineral with the composition BaAl_2_Si_2_O_8_ and a structure consisting of a three-dimensional framework of corner sharing SiO_4_ and AlO_4_ tetrahedra, and Ba^2+^ cations filling interstitial sites. Because of the match in unit-cell dimensions and because the crystal chemistry of paracelsian is closely related to what is expected for sample 4, it is reasonable to assume that the new material is structurally closely related to paracelsian. The ionic radius of K^+^ is 133 pm while that of Ba^2+^ is 134 pm. It therefore makes perfect sense that both cations are able to structurally support the same paracelsian topology, but with Si/Al ratios of 3 and 1, respectively, to maintain charge neutrality.

**Figure 2:**
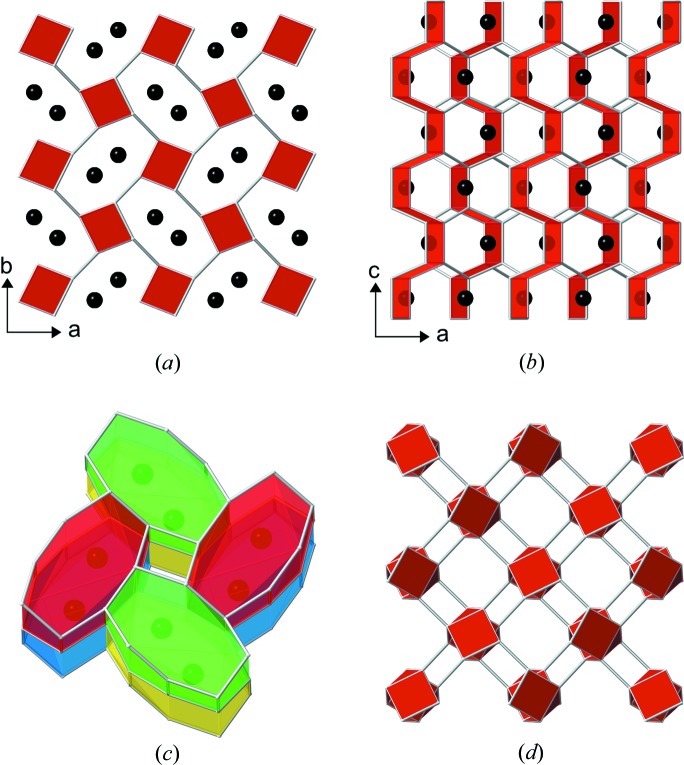
Framework topology of K-paracelsian and a related feldspar represented by connected *T* sites. (*a*) and (*b*) topology of K-paracelsian. Red planes are used to emphasize the double-crankshaft chains and the black spheres represent potassium positions. The structure is shown in projection parallel and perpendicular to the double-crankshaft chains, respectively. (*c*) K-paracelsian cages occupied by potassium atoms. All cages are equivalent by space group symmetry (*Pnam*). Colours have been added to emphasize individual cages. (*d*) Framework topology of the feldspar mineral microcline in projection parallel to the twisted double-crankshaft chains.

A structure model based on the atomic arrangement from orthorhombic (*Pnam*) paracelsian (Smith, 1953 ▸), including the coupled substitution Ba^2+^ + Al^3+^ → K^+^ + Si^4+^, was therefore constructed. A subsequent Rietveld refinement (Coelho, 2018 ▸) using fixed atomic positions and fixed occupancies resulted in a good fit, confirming that the new material has the same framework topology as paracelsian. In the final step of the Rietveld analysis, the fractional coordinates of all atoms and the occupancy of K were refined, resulting in an excellent fit (Fig. 1 ▸) and in meaningful chemical bond distances and angles [see Figs. 2 ▸(*a*)–2(*c*) and the attached CIF]. The refined occupancy of K is 0.98 (1), which is very close to the initially assumed value of 1 for the stoichiometric compound KAlSi_3_O_8_. The name K-paracelsian is proposed for the new material.

The framework topology of common feldspars like orthoclase, albite and microcline as well as most other feldspars consist of twisted interconnected double-crankshaft chains as shown in Fig. 2 ▸(*d*). In contrast, the paracelsian topology consists entirely of straight interconnected double-crankshaft chains, which are also observed in many zeolite topologies such as GIS, APC, MER, PHI, SIV and GME (Baerlocher & McCusker, 2018 ▸).

The question we want to address next is whether K-paracelsian should be considered a feldspar or a zeolite. Feldspar and zeolite topologies consist of corner-connected SiO_4_ and AlO_4_ tetrahedra, forming a periodic three-dimensional structure. Feldspar topologies are made entirely of four, six and eight rings of connected tetrahedra. Some small-pore zeolites such as GIS, APC, MER, PHI and SIV are also constructed entirely from four, six and eight rings, resulting in cages and channels similar in size to those observed in feldspars. In this respect, feldspars could be considered small-pore zeolites. Water content is another property used to define the difference between feldspars and zeolites (Barrer, 1982 ▸): feldspars are anhydrous whereas zeolites are hydrous. For zeolites, it is well known that water can escape along the channels when heated, resulting in a significant change in the observed lattice parameters. For anhydrous feldspars we only expect to observe changes in lattice parameters that are consistent with the thermal expansion properties of the material. *In situ* XRD heating experiments were therefore performed on K-paracelsian and a reference feldspar material.

### 3.3. Comparison with isocompositional microcline   

**Figure 3:**
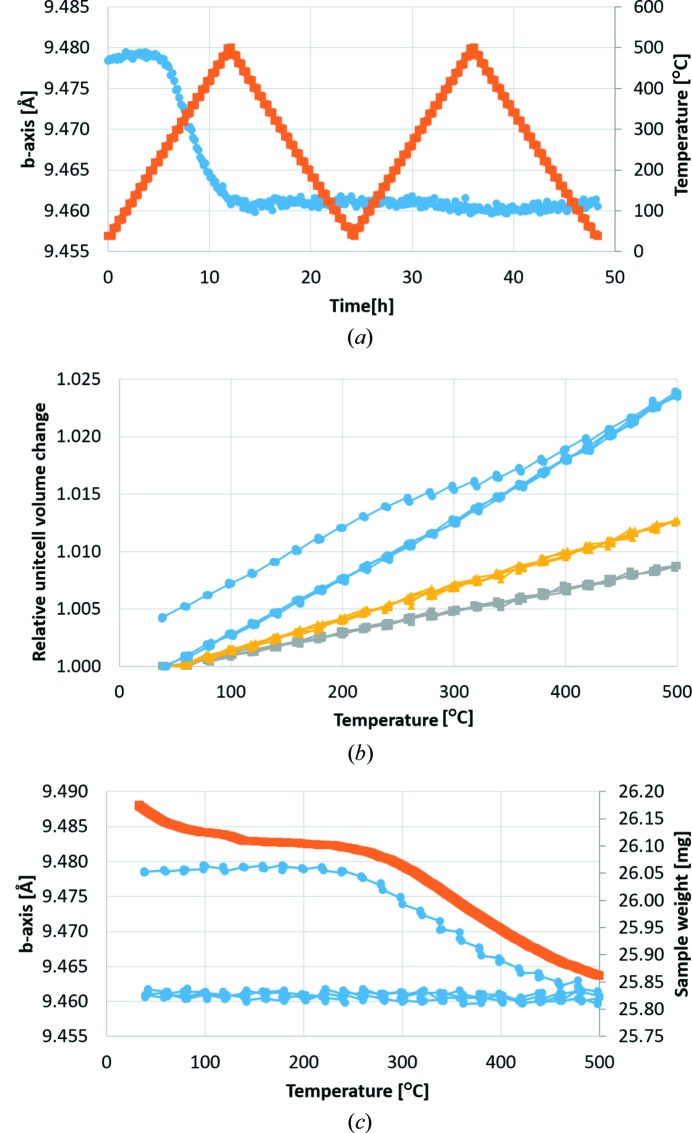
Observed changes in K-paracelsian during two cycles of heating. (*a*) Evolution of the *b* axis (blue) during the temperature programme shown in orange. (*b*) Relative changes in unit-cell volume of K-paracelsian (blue) and the reference feldspars (yellow = albite and grey = microcline). (*c*) Observed sample weight during the TGA experiment (orange) plotted together with observed *b* axis variations (blue) from the *in situ* XRD experiment.

A fragment of microcline from a graphic granite collected in Setesdalen in Norway was used as a reference for the isocompositional K-paracelsian. Both samples were heated in air from room temperature to 500°C in steps of 20°C at a rate of 40°C h^−1^ and cooled to room temperature using a symmetric ramp. XRD data were measured at each step. This heat treatment was repeated twice for each sample [Fig. 3 ▸(*a*), orange data points]. Analysis of the microcline data revealed the sample was composed of 83 wt% microcline and 17 wt% albite. Both feldspars show a simple linear relationship with temperature variation and a fully reversible behaviour on the four temperature ramps as shown in Fig. 3 ▸(*b*). These observations are fully consistent with simple thermal expansion and contraction.

K-paracelsian, however, shows a clear non-linear unit-cell evolution in the temperature range 250–450°C on the first heating ramp [Figs. 3 ▸(*a*) and 3(*b*)]. On the following three ramps, K-paracelsian shows a linear and reversible change similar to that observed in the anhydrous feldspars [Fig. 3 ▸(*b*)]. The observed thermal expansion is dominated by the strong temperature dependence of the *a* and *c* parameters, while the *b* parameter shows almost no temperature dependence on the last three ramps [Fig. 3 ▸(*a*)]. The data from the TGA experiment show a relative mass loss of 5.7% in the temperature range 250–450°C, where the non-linear unit-cell evolution was observed [Fig. 3 ▸(*c*)]. The heat-treated K-paracelsian sample was reanalyzed at room temperature after 4 weeks of exposure to air. The observed lattice parameters were consistent with those of the dehydrated K-paracelsian, indicating that dehydration is not reversible under ambient conditions.

Normal zeolite dehydration behaviour, as observed in chabazite and faujasite, consists of an initial steep gradual weight loss initiated slightly above room temperature and continuing up to 200°C where at least 90% of the water has been evaporated. Most of the remaining water will have been evaporated by 400°C. In contrast to chabazite and faujasite, the zeolite natrolite (Na_2_Al_2_Si_3_O_10_·2H_2_O) shows very little weight loss at temperatures up to 250°C, followed by a steep weight loss of 10% consistent with a loss of both water molecules (Gottardi & Galli, 1985 ▸; van Reeuwijk, 1974 ▸). In contrast to most other zeolites, all water molecules in natrolite are located in relatively well defined positions within the first coordination shell of Na, where they complete a six-fold coordination shell with a slightly distorted trigonal prism geometry (Meier, 1960 ▸).

**Figure 4:**
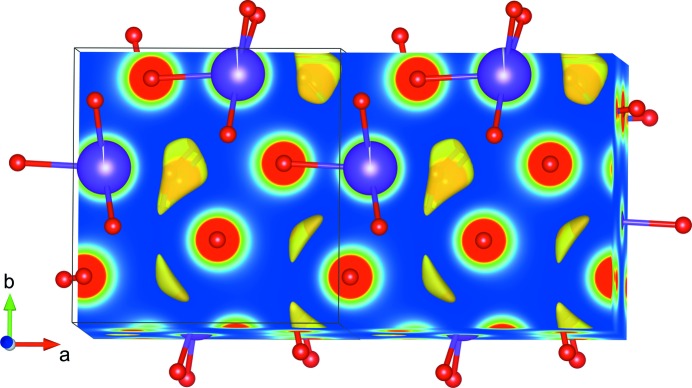
Calculated independent atom model electron density (Momma & Izumi, 2011 ▸) of the K-paracelsian structure. Yellow areas illustrate positions in the cage with minimum electron density.

The dehydration behaviour of K-paracelsian is very similar to that of natrolite, with a weight loss consistent with one water molecule per cage (KAlSi_3_O_8_·H_2_O). Inspection of the calculated independent atom model electron density, as shown in Fig. 4 ▸, reveals a minimum in the density at the position where a water molecule would complete an octahedral coordination of the six oxygen atoms closest to K.

**Figure 5:**
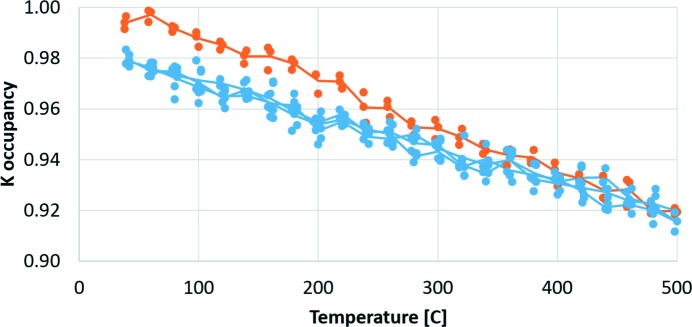
Refined K-occupancies as a function of temperature. Orange points were collected on the first heating ramp.

Inspection of the observed XRD data of the as-synthesized sample did not reveal significant amounts of residual electron density in this position, indicating a more disordered nature of the H_2_O positions. The evolution of the K occupancy as a function of temperature is shown in Fig. 5 ▸. The overall apparent temperature dependence of the K occupancy is an artefact caused by correlation with the displacement parameters. The relative decrease observed on the first heating ramp between 200 and 400°C is reliable and shows that a small fraction of the electron density inside the cages is disappearing at this stage. This observation could be consistent with a water molecule disappearing from the first coordination shell of K, or it could be consistent with an interpretation where a few percent of K ions are missing, leaving space available to be occupied by water molecules. The data are not conclusive on this issue.

## 4. Identification of a simple building scheme of dense double-crankshaft zeolite topologies   

**Figure 6:**
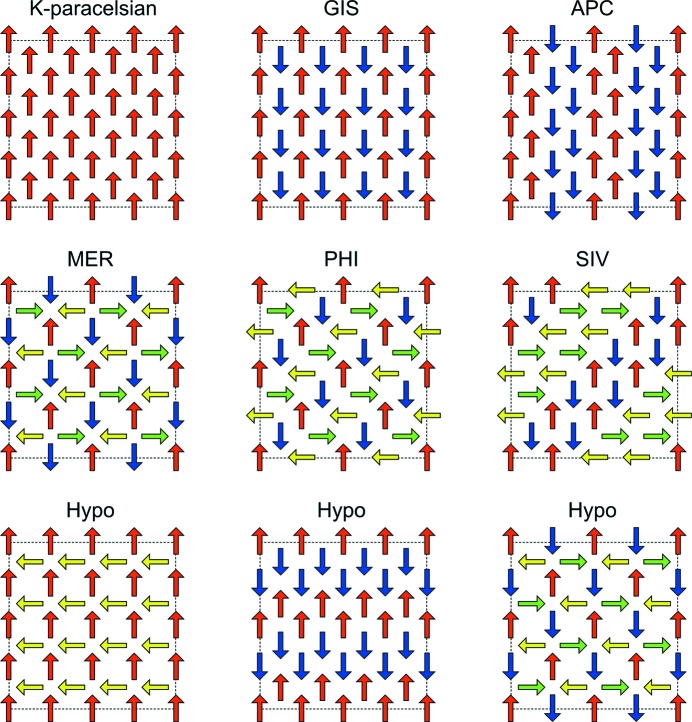
Schematic drawing of the building scheme used to construct the zeolite topologies in the family of dense double-crankshaft topologies. Each arrow represents a double-crankshaft chain, as seen in projection along the chain direction, and the direction of the arrow indicates the relative rotational orientation. There is only one physically meaningful solution to the connectivity between double-crankshaft chains for each of the schematic drawings, which results in the final topology. Colours have been applied for visual emphasis of symmetry.

The topology of K-paracelsian is closely related to the known zeolite topologies GIS, APC, MER, PHI and SIV. These topologies can all be constructed by double-crankshaft motifs placed in a lattice as shown in Fig. 6 ▸. The only difference between these topologies is the relative rotational orientation of individual double-crankshaft chains. GME is another topology that can be constructed entirely of double crankshaft motifs, but in this case, the motifs are arranged in an open hexagonal lattice leading to larger cages and a less dense topology. The building scheme presented in Fig. 6 ▸ defines a family of dense double-crankshaft zeolite topologies, six of which have now been realized. This family consists of topologies with four, six and eight rings and has eight-ring channel systems that range from one-dimensional (K-paracelsian) to two-dimensional (APC) to three-dimensional (GIS, MER, PHI, SIV). A large number of hypothetical topologies can be constructed using the building scheme presented in Fig. 6 ▸, where three of them are shown. There are many approaches to constructing hypothetical zeolite topologies, some of which involve elaborate computational methods (Deem *et al.*, 2009 ▸). Many of these hypothetical topologies may not be possible to realize in practice, because they are thermodynamically unstable. It is worth noting that by applying the above described building scheme, the resulting topologies will be crystallo-chemically healthy in the sense that they all have very similar stabilization energies, including the topologies that have already been realized (GIS, APC, MER, PHI and SIV).

## 5. Conclusions   

K-paracelsian was discovered while screening the hydro­thermal phase space using potassium hydroxide in combination with 1-methyl-4-aza-1-azoniabi­cyclo­[2.2.2]octane hydroxide (1-methyl-DABCO) and the use of an FAU zeolite as the source of Si and Al. The novel synthesized alumino­silicate is compositionally closely related to the mineral microcline and structurally closely related to the mineral paracelsian. Both are part of the feldspar group. In contrast to feldspars, this new material shows intrazeolitic molecular water, which can be extracted upon heating. This suggests that K-paracelsian could be considered a link between feldspars and zeolites.

Furthermore, we have shown that K-paracelsian is the simplest endmember of a family of dense double-crankshaft zeolite topologies. By applying the identified building principle, a number of known zeolite topologies can be constructed. It therefore allowed us to construct a range of hypothetical small-pore topologies that are crystallo-chemically healthy and potentially industrially relevant, which could be realized in future work.

## Supplementary Material

Crystal structure: contains datablock(s) I. DOI: 10.1107/S2052252518016111/ed5017sup1.cif

Structure factors: contains datablock(s) I. DOI: 10.1107/S2052252518016111/ed5017Isup2.hkl

Supplementary figures and tables. DOI: 10.1107/S2052252518016111/ed5017sup3.pdf

CCDC reference: 1878958

## Figures and Tables

**Table 1:** Summarized product phases for the synthesis system FAU–OSDA–KOH

Sample number	Si- and Al- sources	OSDA/Si	KOH/Si	Product phase	*M* (g)/η (%)
1	FAU (Si/Al = 6)	0.2	0.6	LTL + K-paracelsian	0.44/58
2	FAU (Si/Al = 6)	0.1	0.7	LTL + K-paracelsian	0.3/39
3	FAU (Si/Al = 6)	0.2	0.5	ERI + Unknown	0.3/40
4	FAU (Si/Al = 6)	0.2	0.7	K-paracelsian	0.33/45
5	FAU (Si/Al = 6)	0	0.8	LTL + K-paracelsian†	0.28/36
6	ASA (Si/Al = 6)	0.2	0.6	LTL	0.44/58

† Phase only exists in very small amounts.
