# Supplementary material for: K-paracelsian (KAlSi3O8·H2O) and identification of a simple building scheme of dense double-crankshaft zeolite topologies
Source: IUCrJ. 2019 Jan 1;6(Pt 1):66–71. doi: 10.1107/S2052252518016111 (PMC6327177; doi:10.1107/S2052252518016111)
Supplement: Supplementary file 3 [file m-06-00066-sup3.pdf]

IUCrJ

Volume 6 (2019)

Supporting information for article:

K-paracelsian (KAlSi3O8*H2O) and identification of a simple building scheme of dense double-crankshaft zeolite topologies

Cristian-R. Boruntea, Peter N. R. Vennestrøm and Lars F. Lundegaard

S1. Synthesis of 1-methyl-4-aza-1-azoniabicyclo[2.2.2]octane hydroxide (1-methyl-DABCO) used for the preparation of K-Paracelsian.

In a 250 mL flask 20.4 g (0.12 mole) DABCO (Sigma-Aldrich, c = 99 %) was dissolved in 60 mL methanol (Sigma-Aldrich, c = 99 %) and the mixture was stirred for half an hour. The flask was placed on an ice bath and 7.6 mL (0.12 mole) methyl iodide (Sigma-Aldrich, c = 99 %, ρ = 2.28 g/mL) was added dropwise. The ice bath was removed and the reaction mixture was vigorously mixed for 5 days at room temperature. Then, 100 mL diethyl ether (Sigma-Aldrich, c = 99 %) was added and the solid was filtrated and washed with 200 mL diethyl ether. The organic salt was characterized by ^{13}C-NMR, ^{1}H-NMR. The transformation of the iodide salt to the hydroxide form, was done by using the ion exchange Amberlite IRN78 Resin by mixing overnight (24 h). The filtrated solution had a concentration of 12.5 wt. %.

Table S1 Products and chemical composition of selected synthesis using 1-methyl-DABCO from synthesis composition 1.0 SiO_2 : xx Al : aa OSDA : bb NaOH : 20 H_2O crystallized for 7 days at 150 °C.

FAU (Si/Al=12.5)		NaOH	
OSDA	0.45	0.55	0.65
0.1	145/Amorphous	146/Amorphous	147/LEV
0.2	148/Amorphous+ANA	149/LEV+ANA	150/LEV+ANA
0.3	151/LEV+ANA	152/LEV+ANA	153/LEV+ANA
FAU (Si/Al=6)		NaOH	
OSDA	0.5	0.6	0.7
0.1	154/LEV	155/LEV+ANA	156/LEV+ANA
0.2	157/LEV+ANA	158/LEV+ANA	159/LEV+ANA
0.3	160/LEV+ANA	161/LEV+ANA	162/ANA

Table S2 Products and chemical composition of selected synthesis using 1-methyl-DABCO from synthesis composition 1.0 SiO_2 : xx Al : aa OSDA : bb KOH : 20 H_2O crystallized for 7 days at 150 °C, U = Unkown.

Si/Al (FAU/ASA)	OSDA	KOH	SN/PXRD	M (g)/η(%)
15	0.2	0.4	390/Amorphous+U	0.30/41
30	0.2	0.25	391/Amorphous+U	0.35/47
6 (ASA)	0.2	0.6	392/LTL	0.36/40
15 (ASA)	0.2	0.4	393/ERI+Amorphous	0.31/40
30 (ASA)	0.2	0.25	394/Amorphous+U	0.21/28

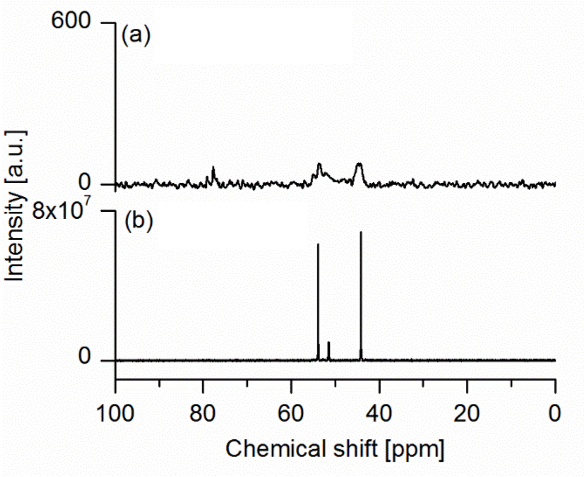

Figure S1 a) ^{13}C CP-MAS NMR of the as-prepared K-Paracelsian zeolite; b) 1D ^{13}C-NMR of the OSDA.

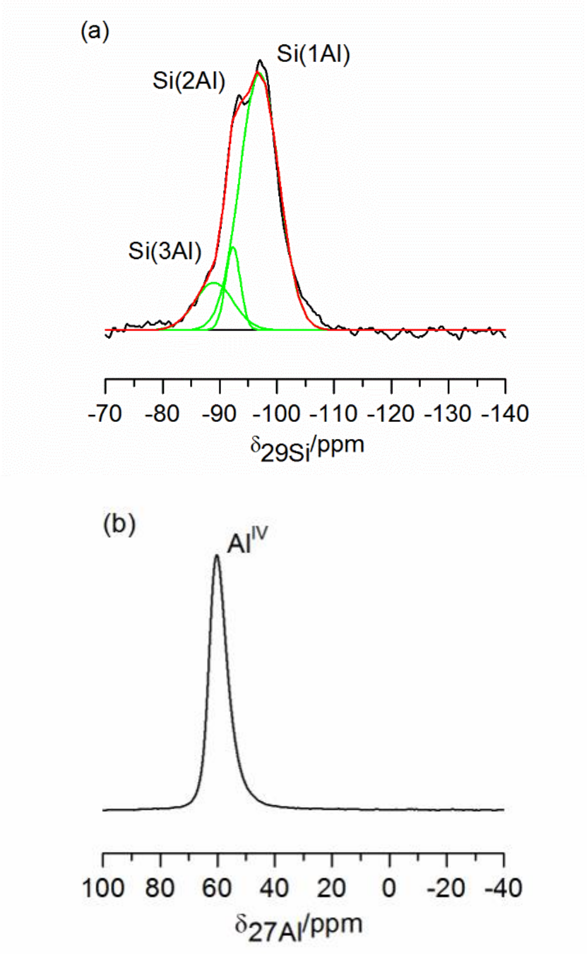

Figure S2 ^{29}Si (a) and ^{27}Al MAS NMR (b) spectra of K-Paracelsian zeolite.
